# Changes in body composition in genetic *C9orf72* carriers: The role of the hypothalamus and thalamus

**DOI:** 10.1002/alz.71669

**Published:** 2026-07-15

**Authors:** Rebekah M. Ahmed, Nga Yan Tse, Martina Bocchetta, Carol Dobson‐Stone, John B. Kwok, John R. Hodges, Muireann Irish, Jonathan R. Rohrer, Olivier Piguet, Glenda Halliday

**Affiliations:** ^1^ Brain and Mind Centre The University of Sydney Sydney Australia; ^2^ Department of Neurology Royal Prince Alfred Hospital Sydney Australia; ^3^ Dementia Research Centre, Department of Neurodegenerative Disease, UCL Queen Square Institute of Neurology University College London London UK; ^4^ School of Medical Sciences The University of Sydney Sydney Australia; ^5^ School of Psychology The University of Sydney Sydney Australia

**Keywords:** FTD, genetic, metabolism, neurodegeneration

## Abstract

**BACKGROUND:**

Patients with sporadic frontotemporal dementia (FTD) display changes in metabolism and body composition. No studies have examined body composition changes in pre‐symptomatic *C9orf72* mutation carriers

**METHODS:**

Asymptomatic *C9orf72* expansion carriers between 2017 and 2022 (*n *= 28 non‐carriers, 16 expansion‐positive), underwent dual‐energy X‐ray absorptiometry (measurement of changes in body composition) and brain magnetic resonance imaging (MRI).

**RESULTS:**

Changes in body composition were identified in the *C9orf72* expansion carrier group compared to non‐carrier group (average 11–12 years prior to onset): lower android‐to‐gynoid ratio (*p* = 0.009), and visceral adipose tissue area (*p *= 0.010). Lower android‐to‐gynoid ratio and lean mass were associated with lower volumes across the cortex, subcortex, and thalamus.

**CONCLUSIONS:**

The current study highlights that genetically at‐risk *C9orf72* patients exhibit altered body composition. These findings align with the growing recognition that changes in genetic frontotemporal dementia/amyotrophic lateral sclerosis (FTD/ALS) extend beyond cognitive/motor symptoms and include early alterations in broader physiological function.

## Highlights

- Genetically at‐risk *C9orf72* patients exhibit altered body composition
- These changes are associated with lower volumes in a distributed network of cortical and subcortical regions including the thalamus
- These changes on average occur a decade before frontotemporal dementia/amyotrophic lateral sclerosis (FTD/ALS) diagnosis
- Changes in genetic FTD/ALS extend beyond cognitive and motor symptoms and include early alterations in broader physiological function

## 1 INTRODUCTION

There is increasing evidence of changes in metabolism, body composition, and eating behaviours in frontotemporal dementia (FTD) and amyotrophic lateral sclerosis (ALS).^1^ Patients with sporadic FTD display abnormal eating behaviors^2^, ^3^, ^4^ and have increased lipid levels,^5^ elevated metabolic rates,^6^ and associated changes in body composition potentially impacting disease progression.^7^ Changes in body composition in sporadic FTD include increased fat mass, visceral adipose tissue and android‐to‐gynoid ratio (increased abdominal fat). These changes have been associated with disturbed neural networks that include key cortical and subcortical structures implicated in autonomic function and limbic processing.^7^

Metabolic changes in FTD have also been related to complex neuroendocrine^8^ and structural alterations affecting the hypothalamus: the latter include diffuse hypothalamic atrophy involving multiple subregions, such as the anterior, posterior, and superior tuberal regions across the FTD and amyotrophic lateral sclerosis (ALS) spectrum.^9^ These changes have also been documented in presymptomatic FTD cohorts including those carrying *MAPT (Microtubule‐ associated protein Tau)* and *C9orf72* mutations, with suggestion of worse atrophy in *MAPT* mutation carriers with tau pathology.^10^, ^11^ Studies also indicate deposition of pathology in the hypothalamus in both sporadic FTD and ALS related to the presence of abnormal eating behavior and sleep changes.^12^

To date, no studies have examined body composition changes in presymptomatic FTD/ALS genetic mutation carriers and their relationship to brain atrophy patterns. This question is of particular relevance for patients along the FTD–ALS spectrum, given the shared genetic and clinical overlap between FTD and ALS.^13^ Furthermore, patients with ALS^13^ show distinct changes in body composition that often occur pre‐symptomatically.^14^ Patients with ALS often have a lower body mass index and lose weight prior to disease onset.^14^ Studies have also shown that patients with ALS have a low baseline percentage body fat and total fat mass and lose body fat with disease progression.^15^ Only one study to our knowledge has explored body composition in ALS, reporting increased visceral fat deposition (abdominal) and loss of subcutaneous fat.^16^ As such, the evidence to date suggests that changes in body composition are common across the ALS–FTD spectrum but may vary depending on clinical phenotype and underlying pathology.

A precise understanding of changes in body composition is critical to expand the physiological phenotyping of presymptomatic cohorts, with these changes potentially affecting diagnosis, disease progression, and the targeting of clinical treatment. To this end, the current study aimed to comprehensively document changes in body composition in a cohort of asymptomatic carriers of genetic abnormalities causal for FTD and ALS compared to a non‐carrier control cohort, using dual‐energy X‐ray absorptiometry (DEXA) scans. This approach offers the distinct advantage of providing objective measures of key body composition metrics including total lean mass, fat mass, android‐to‐gynoid ratio (a measure of the amount of fat around the trunk/abdomen), and visceral adipose tissue (VAT) area (a measure of visceral adipose tissue deposition). To delineate contributing brain regions, changes in body composition were correlated to gray matter volumes of brain regions implicated in metabolism and body composition underpinned by autonomic and limbic functions.

## 2 METHODS

### 2.1 Participants

Probands with autosomal dominant neurodegenerative non‐Alzheimer's dementias and motor abnormalities focused on TAT DNA‐ binding protein 43 (TDP‐43) and α‐synuclein proteinopathies were identified from six large clinic populations in Sydney and Melbourne Australia and a regional brain donor program recruiting from an additional six tertiary referral clinics in Sydney and other New South Wales (NSW)‐based neurologists in the Movement Disorder Society of Australia and New Zealand. Asymptomatic members of these families were recruited between 2017 and 2022 to the National Health and Medical Research Council (NHMRC)‐funded Dominantly‐Inherited Non‐Alzheimer Dementias (DINAD) project (grant #1095127).

All participants underwent genetic counselling prior to study recruitment for genetic testing involving genomic DNA extracted from blood lymphocytes using routine methods. Genetic testing was performed using whole exome sequencing (see gene list under Table S1) as well as polymerase chain reaction (PCR) screening for *C9orf72* repeat expansions^17^ and long‐range PCR^18^ and/or nanopore^19^ screening for *GBA1* mutations. All positive candidate gene mutations were confirmed by Sanger sequencing. Additional study components included clinical and psychometric assessments, collection of blood and other biofluids (cerebrospinal fluid [CSF], saliva, and urine), tests that measure modifiable factors (sleep and core body temperature, movement, metabolism, and food consumption), and structural and functional neuroimaging. Exclusion criteria from the DINAD study included abnormalities on neurological, neuropsychological or psychiatric assessment or contraindications to magnetic resonance imaging (MRI). All assessments were completed within 3 months of MRI acquisition.

#### 2.1.1 Ethics approval

The study was approved by the Human Research Ethics Committee of the Sydney Local Health District (HREC/17/RPAH/6) and all participants provided written informed consent in accordance with the Declaration of Helsinki.

For the present study, families with confirmed TDP‐43 proteinopathies were selected. Those with clinical, psychometric, metabolic, physiological, and structural neuroimaging data were analyzed. The comprehensive standardized assessments consisted of medical and neurological examination, assessments of cognitive and behavioural functioning, clinical interviews, and a structural volumetric brain MRI scan. The physiological component encompassed additional measurement of whole‐body composition (i.e., a DEXA scan) and an informant‐based questionnaire of eating behavior.

### 2.2 Global cognitive, behavioural, and eating behaviour measures

To examine potential contributors to whole‐body composition abnormalities, measures of global cognitive and behavioral functions as well as eating behavior were further administered.

#### 2.2.1 Global cognition

A total of 44 (16 *C9orf72* expansion carriers and 28 non‐carriers) completed the third edition of the Addenbrooke's Cognitive Examination (ACE‐III^20^), a global cognitive screening instrument yielding a total score (max 100; normal score >88) derived from attention, memory, fluency, language, and visuospatial skills subdomains.

#### 2.2.2 Behavioral changes

A total of 35 participants (13 *C9orf72* expansion carriers and 22 non‐carriers) completed the revised version of the Cambridge Behavioural Inventory (CBI‐R^21^). The severity and nature of behavioural symptoms across the following subdomains were analyzed: abnormal behaviour (i.e., behavioural disinhibition), mood changes, odd beliefs (i.e., delusion and hallucinations), abnormal eating habits, sleep, stereotypic behaviors (i.e., perseverative and ritualistic behaviours), and reduced motivation (i.e., apathy and inertia).

#### 2.2.3 Eating behavior

A total of 33 participants (9 *C9orf72* expansion carriers and 24 non‐carriers) completed the self‐report Dietary Questionnaire for Epidemiological Studies Version 3.2 (DQES), providing a comprehensive assessment of average daily intake of foods, energy, and nutrients based on drinking and eating habits in the preceding 12 months. These include average daily intake of energy (including fiber), protein, fat, carbohydrate, and sugars.

**RESEARCH IN CONTEXT**

1. **Systematic review**: A thorough PubMed/literature review using the terms frontotemporal dementia (FTD), amyotrophic lateral sclerosis (ALS), metabolism, hypothalamus, thalamus, body composition autonomic function, and genetic studies was conducted.
2. **Interpretation**: It is increasingly recognized that changes in metabolism and eating behavior occur in neurodegeneration. The current study highlights that genetically at‐risk *C9orf72* patients exhibit altered body composition associated with lower volumes in a distributed network of cortical and subcortical regions including the thalamus on average a decade before FTD/ALS diagnosis. These findings align with the growing recognition that changes in genetic FTD/ALS extend beyond cognitive and motor symptoms and include early alterations in broader physiological function, that are likely mediated by the thalamus with complex interactions between reward and autonomic mechanisms as widely implicated in FTD
3. **Future directions**: Future studies should focus on comparing body composition changes in genetic groups versus sporadic ALS and FTD and the effect that potential treatment of metabolic parameters may have on disease progression and survival. Future studies should also examine the longitudinal changes in body composition and how this may be affected by potential disease modifying therapies and offers the possibility as a potential marker in clinical trials.

### 2.3 Whole‐body composition: DEXA scan

A total of 40 participants (13 *C9orf72* expansion carriers and 27 non‐carriers) completed a single whole‐body scan on a Hologic Horizon A (SN‐300616 M) (Hologic Inc., Bedford, MA www.hologic.com). The Hologic whole‐body scanning dimension was 196 cm by 68 cm, ensuring adequate separation of the arms from the trunk in the whole‐body positioning. DEXA body scan data were analyzed with the QDR system software for Windows (XP) Hologic software APEX 5.6.0.5 (Hologic). Total mass (grams), lean mass (grams), fat mass (grams), and fat percentage were calculated for the whole body and for individual regions of interest: the head, upper limbs, lower limbs, and trunk. Regions of interest were defined as follows: head, immediately below the mandible; trunk, enclosing the chest, midriff, and pelvis; the left and right upper limbs, medial to the head of the humerus; and left and right legs, boundary placed outside of the thigh through to the middle of both legs through the femoral neck and lateral to the pubic ramus. Visceral abdominal fat (VAT area in cm^2^) was initially identified automatically followed by manual adjustment to ensure that the lateral edges of the VAT area were correctly defined.

From the DEXA scan, five key measures including total lean mass; total fat mass; percentile fat (age matched), amount of fat matched to a population of similar age; android‐to‐gynoid ratio, fat in android (trunk area) compared to gynoid area; and VAT area, fat deposited around visceral organs were obtained for each participant.

### 2.4 Neuroimaging

#### 2.4.1 Brain imaging acquisition

All 44 participants (16 *C9orf72* carriers and 28 non‐carriers) underwent volumetric T1‐weighted MRI in a 3T Philips Achieva scanner equipped with a standard 8‐channel head coil using the following parameters: matrix 256 × 256, 200 slices, 1 mm^2^ in‐plane resolution, slice thickness = 1 mm, echo time = 2.6 ms, repetition time = 5.8 ms, flip angle = 8°.

#### 2.4.2 Brain volume analyses

Volumetric MRI scans were bias field corrected and whole‐brain parcellated using the geodesic information flow (GIF) algorithm,^22^ which is based on atlas propagation and label fusion. We combined regions of interest to calculate gray matter volumes of the cortex (orbitofrontal; dorsolateral prefrontal cortex [DLPFC] and ventromedial prefrontal cortex [VMPFC]; motor; frontal pole, opercular, anterior, and posterior insula; temporal pole; superior, lateral, and medial temporal; sensory; medial and lateral parietal; medial and lateral occipital; anterior, middle, and posterior cingulate) and of the subcortical regions (caudate nucleus, accumbens, amygdala, hippocampus, pallidum, putamen, ventral diencephalon, basal forebrain, pons, brainstem).

Given the unique role of the thalamus and hypothalamus in metabolic functions, further segmentation of the thalamus and hypothalamus was performed. Specifically, we used a customized version of the Freesurfer module that accepts the GIF parcellation^23^, ^24^ as input to extract thalamic subregions, which were grouped according to Bocchetta et al. (2020)^23^ and Iglesias et al. (2018)^.^ ^24^ as anteroventral (AV), laterodorsal (LD), lateral posterior (LP), ventral anterior (VA), ventral lateral anterior (VLa), ventral lateral posterior (VLp), ventral posterolateral (VPL), ventromedial (VM), intralaminar, midline, mediodorsal (MD), lateral geniculate (LGN), medial geniculate (MGN), and pulvinar. For the hypothalamus, we applied the segmentation tool of Billot et al. (2020)^25^ leveraging a deep convolutional neural network to extract the following five subunits: (1) anterior inferior (containing supraoptic nucleus); (2) anterior superior (containing the paraventricular nucleus), (3) inferior tuberal (containing the arcuate nucleus, the ventromedial nucleus and the posterior part of the supraoptic nucleus), (4) superior tuberal (containing dorsomedial nucleus, the anterior part of the lateral hypothalamus, and the posterior part of the paraventricular nucleus); and (5) posterior (containing the posterior part of the lateral hypothalamus and the mammillary bodies).

Total intracranial volume (TIV) was computed with Statistical Parametric Mapping 12 (SPM12) software version 6217 (Statistical Parametric Mapping, Wellcome Trust Centre for Neuroimaging, London, UK) running under Matlab R2014a (Math Works, Natick, MA, USA).^26^ Stringent visual checks were conducted on all MRI scans and segmentations to ensure suitable quality (i.e., motion, other imaging artefacts, pathology unlikely to be attributed to FTD or ALS and incorrect anatomic labeling).

### 2.5 Statistical analyses

Data were analyzed using SPSS Statistics, version 24.0 (IBM, Armonk, NY). The statistical significance level was set at *p *< 0.05 for all analyses unless otherwise specified. To derive the demographic, cognitive, behavioral, and metabolic characteristics associated with *C9orf72* repeat expansion, the non‐parametric Mann–Whitney *U* test was used to compare all demographic, cognitive, behavioral, and metabolic variables. Analysis of covariance (ANCOVA) was used to examine differences in brain volumes between the *C9orf72* expansion carrier and non‐carrier groups, while controlling for TIV, age, and sex. A *p*‐value of < 0.05 was regarded as statistically significant. The threshold was not set lower given the small sample size. Categorical variables (i.e., sex) were examined using chi‐square tests.

To identify the potential contribution of brain regions displaying significant between‐group differences to whole‐body composition aberrancies, we examined the associations between significant regional brain volumes and DEXA variables. In order to maximize statistical power, we carried out partial correlations by combining presymptomatic carriers and healthy controls into a single group, with group membership included as a covariate. Statistical significance was set at a more conservative level of *p* ≤ 0.01 to control for Type 1 error.

## 3 RESULTS

### 3.1 Demographics and clinical profile

Demographic variables (i.e., age, sex, body mass index [BMI], proband age at onset, and years to expected age at onset) did not differ significantly across the overall sample (16 *C9orf72* expansion carriers and 28 non‐carriers; Table 1), the DEXA scan subgroup (13 *C9orf72* expansion carriers and 27 non‐carriers; Table 2), or the food intake survey subgroup (9 *C9orf72* expansion carriers and 24 non‐carriers; Table 3). Cognitive profiles were largely comparable between carriers and non‐carriers on the ACE‐III except for a lower language score in the *C9orf72* expansion‐positive group (*p* = 0.031; Table S2). Overall level of behavioral change was also not found to differ significantly between the two groups on the CBI‐R (Table S3).

**TABLE 1:** Demographic characteristics.

	*C9orf72*(*n* = 16)	Non‐carriers(*n* = 28)	*U*	*p*
**Age**	45.19 (13.56)	47.36 (11.16)	202.00	.591
**Sex, F/M**	14/2	18/10	2.766	.096
**BMI**	28.20 (6.95)	30.30 (7.58)	181.00	.294
**Years to expected age at onset**	13.81 (12.06)	–		
**Proband onset age**	59.00 (6.03)	–		

Mean (standard deviation).

Abbreviations: BMI, body mass index; F, female; M, male.

**TABLE 2:** Whole‐body composition characteristics.

	*C9orf72*(*n *= 13)	Non‐carriers(*n *= 27)	*U*	*p*
Age	46.46 (14.43)	47.30 (11.15)	174.00	.965
Sex, F/M	10/3	16/11	1.203^a^	.273
BMI	26.71 (6.86)	30.01 (7.31)	116.00	.086
Years to expected age at onset	12.23 (12.48)	–		
Proband age at onset	58.69 (6.55)	–		
Percentile fat, AM	37.38 (35.75)	52.26 (31.85)	128.00	.170
**Android‐to‐gynoid ratio**	**0.78 (0.22)**	**1.01 (0.22)**	**84.50**	**.009**
**VAT area, cm^2^**	**69.73 (44.32)**	**122.91 (68.25)**	**86.00**	**.010**
Total fat mass, g	24,119.63 (12,577.33)	28,279.04 (12,479.28)	137.00	.266
Total lean mass, g	48,512.62 (9,858.82)	54,603.44 (13,591.85)	129.00	.179

Mean (standard deviation). ^a^Chi‐square value.

Abbreviations: AM , age matched; Android‐to‐gynoid ratio, Fat in android (trunk area) compared to gynoid area; BMI, body mass index; F, female; M, male; VAT area, visceral adipose tissue area.

**TABLE 3:** Group food intake characteristics.

	*C9orf72* (*n *= 9)	Non‐carriers (*n *= 24)	*U*	*p*
Age	46.11 (15.25)	49.79 (11.66)	90.00	.486
Sex, F/M	9/0	17/7	3.332^a^	.068
BMI	27.14 (7.00)	31.06 (7.86)	73.00	.157
Years to expected age at onset	11.00 (12.25)	–		
Proband age at onset	57.11 (3.86)	–		
Total energy including fiber, kJ/day	7,311.53 (1,403.75)	8,129.55 (2,451.07)	87.00	.396
Total carbohydrate, g/day	144.01 (52.64)	153.89 (49.84)	90.00	.467
Total sugars, g/day	75.35 (33.48)	73.92 (31.05)	106.00	.936
Food protein, g/day	78.58 (16.18)	85.63 (28.93)	101.00	.777
Food fat, g/day	78.93(13.30)	91.65(34.32)	96.00	.628

Food indices excluded alcohol intake (i.e., food intake only), whereas total indices included alcohol intake (i.e., food intake + alcohol intake).

Mean (standard deviation). ^a^Chi‐square value.

Abbreviations: BMI, body mass index; DQES, Dietary Questionnaire for Epidemiological Studies; F,  female; M, male.

### 3.2 Metabolic profile associated with *C9orf72* status

As shown in Table 2, a distinct whole‐body composition profile was identified in the *C9orf72* expansion carrier relative to the non‐carrier group, characterized by significantly lower android‐to‐gynoid ratio (*p* = 0.009) and VAT area (*p *= 0.010). No differences were found in total lean mass, total fat mass, or age‐matched fat percentile between *C9orf72* carriers and non‐carriers (Table 2). Notably, average total daily intake of energy (including fiber), carbohydrate, and sugars did not differ between groups (all *p*‐values > 0.05; Table 3).

### 3.3 Neuromaging deficits associated with *C9orf72* status

After correcting for age, sex, and TIV, *C9orf72* expansion carriers demonstrated significantly lower volumes in distributed cortical brain regions when compared to non‐carriers. These included the frontal pole, orbitofrontal, VMPFC, motor, anterior cingulate, middle cingulate, posterior insular, medial parietal, and medial and lateral temporal cortex (all *p*‐values < 0.05; Table S4, note lower *p*‐value threshold not used due to small sample size). Volume of subcortical regions including the bilateral caudate, left pallidum, and right nucleus accumbens and amygdala was also significantly lower in the *C9orf72* expansion carrier relative to the non‐carrier group (all *p*‐values < 0.05; Table S5).

We further examined the potential subregional volume differences in thalamic and hypothalamic subregions. Relative to non‐carriers, the *C9orf72* expansion‐positive group demonstrated significantly lower thalamic volumes within the right LGN as well as the left VLp and MD (all *p*‐values < 0.05; Table S6). Within the hypothalamus, significantly lower volumes emerged for bilateral inferior tuberal and left superior tuberal subregions in *C9orf72* carriers (all *p*‐values < 0.05; Table S7).

### 3.4 Correlations between brain volumes and metabolic measures

Partial correlations were then run across the entire cohort to explore associations between brain regions showing significant between‐group differences and DEXA variables, accounting for the effect of group membership.

**FIGURE 1:**
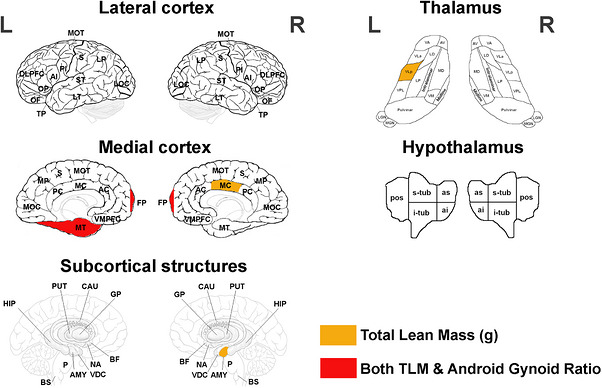
Brain regional volumes associated with body composition measures. Brain renderings depicting regions showing significant positive associations between gray matter volume and android‐to‐gynoid ratio and total lean mass. Statistical maps are thresholded at *p* ≤ 0.01. ai, anterior inferior; AMY, amygdala; as, anterior superior; AV, anteroventral; BF, basal forebrain; BS, brainstem; CAU, caudate; GP, pallidum; HIP, hippocampus; i‐tub, tuberal inferior; LD, laterodorsal; LGN, lateral geniculate; LP, lateral posterior; MD, mediodorsal; MGN, medial geniculate; NA, nucleus accumbens; P, pons; pos, posterior; PUT, putamen; s‐tub, tuberal superior; TLM, total lean mass; VA, ventral anterior; VDC, ventral diencephalon; VLa, ventral lateral anterior; VLp, ventral lateral posterior; VM, ventromedial; VPL, ventral posterolateral.

Lower android‐to‐gynoid ratio and lean mass were associated with lower volumes of primarily the medial temporal and middle cingulate cortices, whereas negative association was identified for frontal pole volumes (all *p*‐values ≤.01; Figure 1; Table S8).

Subcortical involvement was selectively observed for total lean mass, implicating the amygdala (*p* = 0.008) with nucleus accumbens approaching significance (*p* = 0.019; Figure 1; Table S9). Notably, gray matter volume of the VLp subregion of the thalamus was associated total lean mass (*p* = 0.002; Figure 1; Table S10). No significant association was identified with the hypothalamic regions (Table S11).

## 4 DISCUSSION

**FIGURE 2:**
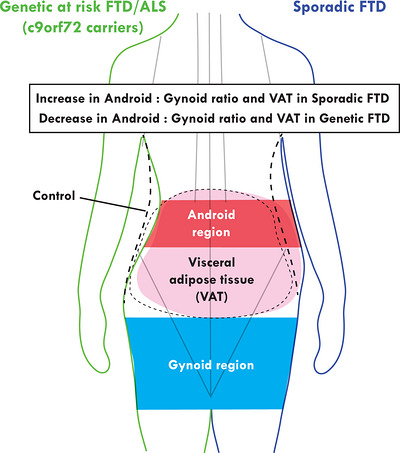
Proposed differences in body composition profile in genetic at‐risk FTD/ALS (*C9orf72* expansion carriers) relative to sporadic FTD. Distinct android‐to‐gynoid ratio and visceral adipose tissue deposition alterations in genetic (green) and sporadic (purple) FTD and controls (Black). ALS, amyotrophic lateral sclerosis; FTD, frontotemporal dementia; VAT, visceral adipose tissue.

The current study shows changes in body composition in presymptomatic at‐risk carriers of the *C9orf72* repeat expansion compared to non‐carriers. These changes occurred on average 11–12 years prior to predicted disease onset and were characterized by reduced android‐to‐gynoid ratio and visceral adipose tissue deposition. These results suggest reduced fat deposition abdominally around a decade before symptom onset, aligning with past ALS research that show reduced BMI and fat deposition 5–10 years preceding disease onset.^27^ Of interest, these results also directly contrast findings in sporadic FTD, which tended to report an increased android‐to‐gynoid ratio and VAT fat deposition,^7^ suggesting that these two conditions may have diverse metabolic profiles related to the underlying genetic mutations and potential co‐presentation with ALS (see Figure 2 for a visual illustration).

Previous sporadic ALS studies have shown that patients develop decreases in BMI up to 10 years pre‐diagnosis, but prior to that have an increased BMI potentially as a compensatory mechanism.^14^ The current study found no changes in BMI between at‐risk carriers and their non‐carrier controls, but definite changes in body composition with reduced fat storage. This is potentially related to a hypermetabolic state affecting fat storage and body composition. A hypermetabolic state has been found in both ALS and FTD patients,^6^ with findings of increased resting energy expenditure by 10% in ALS cases compared to healthy controls^28^, ^29^ and further mouse model evidence of this occurring presymptomatically.^30^ This hypermetabolic state has been linked to hypothalamic changes, with atrophy of the hypothalamus found in both ALS patients and pre‐symptomatic ALS mutation carriers.^31^ Hypothalamic atrophy has also been extensively shown in sporadic and genetic FTD patients both in imaging^8^, ^9^, ^32^, ^33^ and pathological^12^ studies and related to changes in metabolism and eating behavior.

Our cohort of presymptomatic patients also showed significant atrophy involving a distributed network of brain regions across predominantly frontal (frontal pole, motor, orbitofrontal, and VMPFC), temporal (medial and lateral temporal cortex), anterior and middle cingulate, and posterior insular cortex. In terms of the subcortical regions, significantly lower volumes localized to bilateral caudate, left pallidum, and right accumbens and amygdala were also evident in the *C9orf72* expansion‐positive group. In line with our observations, these regions have been shown previously to be involved in *C9orf72* mutation carriers^11^, ^34^ and also FTD and related to eating and reward changes.^4^

Notably, we also identified atrophy involving the hypothalamus, particularly left inferior and superior tuberal and right inferior tuberal hypothalamus in *C9orf72* mutation carriers. Involvement of the hypothalamus has been shown previously in both sporadic and genetic FTD, with hypothalamic atrophy and pathological deposition of TDP‐43 in behavioral variant FTD and ALS^12^, ^35^ related to abnormal eating behavior and changes in metabolic hormones.^32^ Previously we have shown that across the ALS–FTD spectrum, lower volumes of the anterior inferior and superior and the posterior subregion of the hypothalamus were associated with reduced motivation (i.e., apathy and inertia) in addition to eating habit changes.^9^ In ALS, atrophy of the posterior hypothalamus has been related to weight loss.^36^ In genetic FTD, hypothalamic atrophy has been shown across all gene groups, with the greatest reductions in *MAPT* mutation carriers involving the anterior superior, posterior, and superior and inferior tuberal regions. Similar patterns of atrophy were seen in *C9orf72* and *GRN* mutation carriers, but to a lesser extent than *MAPT* mutation carriers.^33^ Of note, atrophy in all regions in the previous study, apart from the inferior tuberal region, correlated to abnormal eating behavior.^33^ Convergently, the current study also showed the greatest atrophy in both the inferior and superior tuberal regions in *C9orf72* mutation carriers compared to non‐carriers. The tuberal region is known to play a role in controlling metabolism and eating behavior, as it includes the ventromedial, dorsomedial, and arcuate nuclei. These nuclei contain key neurons that are involved in mediating metabolism and energy intake including those containing Pro‐opiomelanocortn (POMC) and Cocaine‐ and Amphetamine Regulated Trasncript (CART) which are anorexigenic and others containing Neuropeptide Y/ Agouti Related peptide (NPY/AgRP), which are orexigenic.^37^ As such, further studies are required to delineate the interaction between these specific neuronal types, hormonal levels, and body composition in at‐risk genetic *C9orf72* carriers.

The thalamus also likely plays a role in changes in body composition and is well known to be involved in FTD,^38^, ^39^ with *C9orf72* expansion carriers having the greatest degree of atrophy.^40^ In the current study compared to non‐carriers, *C9orf72* expansion carriers demonstrated significantly lower thalamic volumes across the LGN, VLp, and MD subregions, suggesting significant thalamic involvement prior to symptom onset.

Lower android‐to‐gynoid ratio and lean mass were significantly associated with abnormal volumes across the cortex (predominantly the frontal pole, medial temporal, and middle cingulate cortices), as well as the subcortex anchored in the amygdala. Notably, thalamic involvement (gray matter volume of the VLp subregion of the thalamus) was also observed for total lean mass. These findings suggest that in genetically at‐risk *C9orf72* carriers changes in body composition may be related to a distributed neural network subserving limbic and autonomic function, with known connections between the thalamus, and core limbic structures centering on the amygdala.^41^ Our results therefore point to a strong interplay between disrupted limbic function affecting eating behaviour coupled with autonomic dysregulation influencing body composition. Notably, the current study found no changes in energy intake in the at‐risk genetic carriers, suggesting that the observed changes in body composition are not simply secondary to altered intake. Rather, these findings converge with past findings to point toward underlying physiological and hormonal changes, warranting further investigation to ascertain the nature of these interactions.

Arguably, a limitation to the current study is the relatively small sample size, including a lack of *MAPT* and *GRN* mutation carriers. Studies with larger sample sizes will be needed to confirm these trends. Given the number of statistical tests run in the brain region analyses without multiple comparison correction due to small sample size, caution should be taken when interpreting the robustness of the results. Validation in a larger, external cohort will be invaluable, although this may prove challenging given the rarity of the conditions and genetic mutations. The current study would also benefit from longitudinal follow‐up to track changes in body composition and food intake to elucidate whether these findings remain as the neurodegenerative process takes hold. Finally, the current results are in contrast to body composition changes in sporadic FTD, which showed an increased android‐to‐gynoid ratio, VAT, and fat mass.^7^ This suggests that sporadic FTD may potentially have metabolic markers that are different from presymptomatic genetically at‐risk patients, that may be dependent on whether patients develop FTD versus ALS. This hypothesis will need to be verified with the inclusion of a sporadic FTD and ALS groups in future studies and longitudinal studies recording presymptomatic patients and their eventual phenotype.

## 5 CONCLUSION

The current study highlights that genetically at‐risk *C9orf72* patients exhibit altered body composition associated with lower volumes in a distributed network of cortical and subcortical regions including the thalamus. These findings align with the growing recognition that changes in genetic FTD and ALS extend beyond cognitive and motor symptoms and include changes in broader physiological function, that are likely mediated by the thalamus with complex interactions between limbic and autonomic mechanisms as widely implicated in FTD. Together, these novel findings offer new markers that may aid in earlier diagnosis, track disease progression, and potentially serve as potential treatment targets to slow or prevent disease onset.

## CONFLICT OF INTEREST STATEMENT

The authors declare no conflicts of interest. Author disclosures are available in the Supporting Information.

## CONSENT STATEMENT

All participants provided informed consent to participate in the study

## Supporting information




Supporting Information

Supporting Information

Supporting Information
