# Supplementary material for: Changes in body composition in genetic C9orf72 carriers: The role of the hypothalamus and thalamus
Source: Alzheimers Dement. 2026 Jul 15;22(7):e71669. doi: 10.1002/alz.71669 (PMC13370791; doi:10.1002/alz.71669)
Supplement: Supplementary file 2 — Supporting Information [file ALZ-22-e71669-s001.docx]

| **Supplementary Table 2. Group Differences in Cognitive Functions** | | | | | |
| --- | --- | --- | --- | --- | --- |
|  | ***C9orf72***  **(*n* = 16)** | **Non-carriers**  **(*n* = 28)** | ***GRN***  ***(n=4)*** | ***U*** | ***p*** |
| ACE-III Total | 89.69(18.57) | 90.00(19.42) | 97.25(3.59) | 189.5 | 0.398 |
| Attention | 17.29(1.07)* | 17.00(1.10)* | 17.25(0.96) | 148.5 | 0.308 |
| Verbal Fluency | 11.94(1.61) | 12.32(1.39) | 13.25(0.96) | 195 | 0.469 |
| Language | 23.27(5.99)** | 23.96(6.23) | 26.00(0.00) | 133 | 0.031 |
| Memory | 24.57(1.45)* | 24.69(1.19)* | 25.00(2.00) | 178.5 | 0.918 |
| Visuospatial | 15.19(2.26) | 15.00(2.13) | 15.75(0.50) | 201 | 0.496 |

Mean (Standard Deviation).

ACE-III = Addenbrooke's Cognitive Examination (third edition).

*Two patients with missing data.

**One patient with missing data.

| **Supplementary Table 3. Group Differences in behavioral characteristics** | | | | | |
| --- | --- | --- | --- | --- | --- |
|  | ***C9orf72***  **(*n* = 13)** | **Non-carriers**  **(*n* = 22)** | ***GRN***  ***(n=2/3)*** | ***U*** | ***p*** |
| Memory | 8.51(7.74) | 12.50(21.08) | 19.79(10.05) | 142.5 | 0.986 |
| Everyday skills | 3.08(6.93) | 2.27(6.12) | 1.67(2.89) | 126.5 | 0.443 |
| Self-Care | 1.92(6.93) | 1.42(5.43) | 2.08(3.61) | 141.5 | 0.916 |
| Abnormal Behaviour | 3.85(7.31) | 6.06(8.21) | 30.56(33.68) | 116.5 | 0.327 |
| Mood | 10.90(9.48) | 8.52(10.83) | 9.38(4.42) | 114.5 | 0.311 |
| Beliefs | 1.92(6.93) | 1.14(3.90) | 0.00(0.00) | 142 | 0.944 |
| Eating Habits | 5.13(7.73) | 3.13(5.71) | 9.38(13.26) | 124.5 | 0.467 |
| Sleep | 16.35(19.35) | 19.89(22.71) | 12.50(0.00) | 132.5 | 0.708 |
| Stereotypic and motor behaviours | 9.62(10.40) | 9.38(11.53) | 21.88(22.10) | 135.5 | 0.789 |
| Motivation | 5.26(11.09) | 11.36(17.20) | 15.00(21.21) | 106 | 0.153 |

Mean (Standard Deviation).

CBI-R = The Cambridge Behavioural Inventory-Revised.

| **Supplementary Table 4. Group Differences in Cortical Regional Volumes** | | | | | |
| --- | --- | --- | --- | --- | --- |
|  | **C9orf72**  **(*n* = 16)** | **Non-carriers**  **(*n* = 28)** | ***GRN***  ***(n=4)*** | ***F*** | ***p*** |
| **Orbitofrontal** |  |  |  |  |  |
| Left | 9,918.71(1,000.19) | 10,259.19(1,113.27) | 10,363.50(902.53) | 1.299 | .261 |
| Right | 9,768.83(756.11) | 10,405.90(1,178.79) | 10,010.31(786.65) | 5.475 | .025 |
| **DLPFC** |  |  |  |  |  |
| Left | 39,006.55(4,964.12) | 40,044.49(5,919.78) | 39,596.37(2,254.33) | .759 | .389 |
| Right | 38,144.06(4,304.72) | 39,448.13(5,662.64) | 38,562.75(2,124.35) | .985 | .327 |
| **VMPFC** |  |  |  |  |  |
| Left | 9,235.01(1,014.45) | 9,608.69(1,046.59) | 9,773.37(1,067.23) | 3.774 | .059 |
| Right | 11,035.72(1,218.71) | 11,539.01(1,137.08) | 11,770.40(1,088.26) | 4.171 | .048 |
| **Motor** |  |  |  |  |  |
| Left | 18,121.81(2,000.09) | 19,779.08(2,444.25) | 19,205.35(2,669.17) | 6.063 | .018 |
| Right | 18,480.83(1,899.85) | 19,480.98(2,666.20) | 19,279.07(2,495.19) | 1.522 | .225 |
| **Opercular** |  |  |  |  |  |
| Left | 5,376.35(692.26) | 5,543.97(936.68) | 5,295.08(910.79) | .144 | .706 |
| Right | 5,299.67(775.46) | 5,359.22(758.91) | 5,225.32(782.41) | .008 | .929 |
| **Frontal Pole** |  |  |  |  |  |
| Left | 1,384.95(372.14) | 1,617.07(602.39) | 1,760.54(474.04) | 7.828 | .008 |
| Right | 1,680.14(358.21) | 1,967.22(619.41) | 2,246.24(348.90) | 8.785 | .005 |
| **Medial Temporal** |  |  |  |  |  |
| Left | 13,380.61(1,369.08) | 14,051.37(1,798.68) | 13,121.57(1,490.37) | .387 | .019 |
| Right | 13,435.74(1,361.04) | 13,959.51(1,547.60) | 13,635.79(1,408.22) | .226 | .637 |
| **Lateral Temporal** |  |  |  |  |  |
| Left | 31,983.82(2,849.84) | 33,456.84(4,099.52) | 31,838.36(1,916.43) | 2.242 | .142 |
| Right | 31,219.36(2,864.70) | 33,724.81(4,182.94) | 32,715.79(3,824.14) | 7.985 | .007 |
| **Temporal Pole** |  |  |  |  |  |
| Left | 6,937.50(786.92) | 7,278.72(1,410.19) | 6,980.04(135.14) | .003 | .955 |
| Right | 7,080.69(746.72) | 7,229.83(1,419.74) | 7,586.59(1,269.84) | 1.453 | .235 |
| **Supra Temporal** |  |  |  |  |  |
| Left | 5,364.17(915.53) | 5,538.50(798.50) | 5,583.54(1,005.47) | .011 | .917 |
| Right | 4,641.47(631.52) | 4,770.65(632.53) | 4,742.34(716.82) | .334 | .566 |
| **Medial Parietal** |  |  |  |  |  |
| Left | 8,174.19(1,279.51) | 8,841.08(1,350.33) | 7,928.02(659.90) | 2.996 | .091 |
| Right | 7,949.69(1,118.33) | 8,874.40(1,401.65) | 8,396.28(1,184.70) | 7.118 | .011 |
| **Lateral Parietal** |  |  |  |  |  |
| Left | 23,432.94(3,089.50) | 25,067.77(3,350.34) | 23,043.74(2,851.69) | 3.384 | .073 |
| Right | 22,543.49(3,044.57) | 24,177.91(2,783.46) | 22,666.79(2,788.79) | 3.427 | .072 |
| **Sensory** |  |  |  |  |  |
| Left | 9,573.89(1,421.59) | 9,867.37(1,290.89) | 9,253.77(1,690.88) | .207 | .651 |
| Right | 8,454.50(1,208.85) | 9,143.18(1,147.32) | 7,879.12(1,620.60) | 3.928 | .055 |
| **Medial Occipital** |  |  |  |  |  |
| Left | 16,158.82(2,693.94) | 17,557.69(2,933.67) | 16,420.98(1,629.00) | 1.944 | .171 |
| Right | 15,604.13(2,782.12) | 17,278.90(3,306.56) | 15,977.53(884.38) | 1.978 | .168 |
| **Lateral Occipital** |  |  |  |  |  |
| Left | 12,978.17(2,217.33) | 13,972.15(2,188.87) | 13,675.29(1,942.21) | 2.582 | .116 |
| Right | 11,662.27(2,115.23) | 12,825.83(2,145.91) | 11,893.56(999.61) | 3.388 | .073 |
| **Anterior Cingulate** |  |  |  |  |  |
| Left | 5,174.54(611.83) | 5,640.40(965.67) | 5,359.50(471.68) | 5.458 | .025 |
| Right | 3,928.53(658.30) | 4,266.40(753.36) | 3,964.91(567.43) | 2.473 | .124 |
| **Middle Cingulate** |  |  |  |  |  |
| Left | 4,269.47(471.68) | 4,529.86(888.58) | 4,129.33(293.11) | .918 | .344 |
| Right | 4,457.41(682.10) | 4,938.15(853.15) | 4,455.52(170.97) | 4.123 | .049 |
| **Posterior Cingulate** |  |  |  |  |  |
| Left | 4,587.91(716.31) | 4,804.18(981.73) | 4,437.87(932.11) | .048 | .828 |
| Right | 4,271.54(752.00) | 4,585.18(859.92) | 4,129.38(735.34) | 1.232 | .274 |
| **Anterior Insula** |  |  |  |  |  |
| Left | 3,410.71(437.62) | 3,587.32(481.63) | 3,825.87(346.26) | 1.469 | .233 |
| Right | 3,452.21(395.31) | 3,699.90(502.56) | 3,692.16(341.98) | 2.845 | .100 |
| **Posterior Insula** |  |  |  |  |  |
| Left | 1,581.01(170.37) | 1,730.11(266.48) | 1,690.36(311.26) | 3.931 | .054 |
| Right | 1,578.82(191.39) | 1,744.11(279.15) | 1,831.84(291.55) | 4.472 | .041 |

Mean (Standard Deviation).

BMI = body mass index; DLPFC = dorsolateral prefrontal cortex; VMPFC = ventromedial prefrontal cortex.

| **Supplementary Table 5. Group Differences in Subcortical Volumes** | | | | | |
| --- | --- | --- | --- | --- | --- |
|  | ***C9orf72***  **(*n* = 16)** | **Non-carriers**  **(*n* = 28)** | ***GRN***  ***(n=4)*** | ***F*** | ***p*** |
| **Pallidum** |  |  |  |  |  |
| **Left** | 1,733.39(219.43) | 1,847.93(189.20) | 1,902.81(159.54) | 4.672 | .037 |
| **Right** | 1,760.26(197.82) | 1,868.97(198.97) | 1,869.82(158.94) | 3.957 | .054 |
| **Putamen** |  |  |  |  |  |
| **Left** | 3,920.03(478.56) | 4,172.65(486.85) | 4,219.08(308.84) | 3.919 | .055 |
| **Right** | 3,875.97(413.73) | 4,116.11(508.84) | 4,029.11(284.04) | 3.743 | .060 |
| **Ventral Diencephalon** |  |  |  |  |  |
| **Left** | 4,295.22(446.77) | 4,450.29(500.30) | 4,450.05(498.75) | .279 | .601 |
| **Right** | 4,212.91(439.12) | 4,383.61(502.50) | 4,307.05(448.28) | .438 | .512 |
| **Nucleus Accumbens** |  |  |  |  |  |
| **Left** | 505.69(68.72) | 533.68(70.08) | 540.70(47.09) | 1.123 | .296 |
| **Right** | 471.69(52.41) | 507.64(66.48) | 492.70(30.74) | 5.052 | .030 |
| **Amygdala** |  |  |  |  |  |
| **Left** | 1,616.01(142.22) | 1,720.29(217.95) | 1,634.61(240.09) | 2.090 | .156 |
| **Right** | 1,573.82(195.13) | 1,697.14(199.19) | 1,606.86(206.44) | 4.252 | .046 |
| **Caudate** |  |  |  |  |  |
| **Left** | 2,733.15(274.38) | 2,980.68(411.88) | 2,969.46(194.22) | 5.967 | .019 |
| **Right** | 2,880.59(186.27) | 3,178.61(467.05) | 3,056.95(149.19) | 7.955 | .008 |
| **Hippocampus** |  |  |  |  |  |
| **Left** | 3,568.15(322.02) | 3,716.36(385.29) | 3,717.90(337.49) | 1.283 | .264 |
| **Right** | 3,572.97(326.81) | 3,831.15(415.47) | 3,943.39(390.52) | 3.696 | .062 |
| **Basal Forebrain** |  |  |  |  |  |
| **Left** | 479.88(44.97) | 488.61(62.80) | 497.46(50.30) | .051 | .823 |
| **Right** | 505.75(54.38) | 530.36(71.14) | 511.95(62.18) | 1.725 | .197 |
| **Brainstem** | 7,176.50(524.77) | 7,580.36(739.23) | 7,432.73(522.67) | 2.008 | .164 |
| **Pons** | 9,345.45(1,022.81) | 9,839.36(1,300.04) | 9,395.21(1,451.73) | .496 | .485 |

Mean (Standard Deviation).

| **Supplementary Table 6. Group Differences in Thalamic Subregional Volumes** | | | | | | | | | |  |  |
| --- | --- | --- | --- | --- | --- | --- | --- | --- | --- | --- | --- |
|  | ***C9orf72***  **(*n* = 16)** | **Non-carriers**  **(*n* = 28)** | | ***GRN***  ***(n=4)*** | ***F*** | | | ***p*** |  |  |  |
| **Thalamus** |  |  | |  |  | | |  |  |  |  |
| **Left** | 5391.76(852.76) | 6115.67(1160.91) | | 5575.86(547.50) | 3.010 | | | .091 |  |  |  |
| **Right** | 5187.43(827.28) | 5915.84(1102.83) | | 5381.72(368.11) | 3.300 | | | .077 |  |  |  |
| **MGN** |  |  | |  |  | | |  |  |  |  |
| **Left** | 102.62(31.48) | 112.52(31.42) | | 107.58(34.15) | .408 | | | .527 |  |  |  |
| **Right** | 97.69(28.22) | 108.30(35.12) | | 100.80(34.40) | .636 | | | .430 |  |  |  |
| **LGN** |  |  | |  |  | | |  |  |  |  |
| **Left** | 130.23(30.32) | 151.02(34.13) | | 142.49(14.50) | 3.281 | | | .078 |  |  |  |
| **Right** | 130.94(32.60) | 150.70(34.63) | | 147.19(23.69) | 4.609 | | | .038 |  |  |  |
| **VPL** |  |  | |  |  | | |  |  |  |  |
| **Left** | 689.52(101.11) | 787.15(148.03) | | 757.36(112.01) | 3.291 | | | .077 |  |  |  |
| **Right** | 640.77(83.67) | 735.17(159.13) | | 668.00(59.83) | 2.482 | | | .123 |  |  |  |
| **VLa** |  |  | |  |  | | |  |  |  |  |
| **Left** | 553.35(66.55) | 634.74(126.57) | | 546.05(65.08) | 3.530 | | | .068 |  |  |  |
| **Right** | 547.86(75.51) | 619.76(121.62) | | 552.81(38.88) | 2.394 | | | .130 |  |  |  |
| **VM** |  |  | |  |  | | |  |  |  |  |
| **Left** | 16.24(2.46) | 18.07(3.96) | | 16.35(2.97) | 1.087 | | | .303 |  |  |  |
| **Right** | 15.13(2.34) | 17.18(4.56) | | 14.55(1.69) | 1.016 | | | .320 |  |  |  |
| **AV** |  |  | |  |  | | |  |  |  |  |
| **Left** | 112.83(26.81) | 123.64(38.92) | | 102.70(12.83) | .140 | | | .711 |  |  |  |
| **Right** | 114.42(29.96) | 128.77(39.75) | | 111.51(15.58) | .260 | | | .613 |  |  |  |
| **VLp** |  |  | |  |  | | |  |  |  |  |
| **Left** | 697.18(80.28) | 799.37(141.33) | | 711.97(69.55) | 5.146 | | | .029 |  |  |  |
| **Right** | 683.63(78.26) | 772.81(137.65) | | 699.66(41.68) | 3.299 | | | .077 |  |  |  |
| **LP** |  |  | |  |  | | |  |  |  |  |
| **Left** | 103.20(18.58) | 118.87(25.60) | | 108.80(5.94) | 2.613 | | | .114 |  |  |  |
| **Right** | 93.13(16.40) | 107.73(26.08) | | 101.98(14.70) | 2.129 | | | .153 |  |  |  |
| **LD** |  |  | |  |  | | |  |  |  |  |
| **Left** | 26.35(9.88) | 29.43(14.03) | | 23.41(7.53) | .160 | | | .691 |  |  |  |
| **Right** | 23.38(8.17) | 27.22(14.42) | | 21.76(10.36) | .248 | | | .621 |  |  |  |
| **VA** |  |  | |  |  | | |  |  |  |  |
| **Left** | 401.57(63.74) | 448.74(82.88) | | 388.46(45.26) | 2.015 | | | .164 |  |  |  |
| **Right** | 397.03(72.50) | 443.31(81.02) | | 415.01(27.70) | 1.831 | | | .184 |  |  |  |
| **Midline** |  | |  |  |  |  | | | | |  |
| **Left** | 18.84(6.38) | | 20.49(5.85) | 17.53(4.35) | .145 | | .705 | | | | |
| **Right** | 17.28(6.17) | | 19.96(6.67) | 17.78(5.33) | .777 | | .384 | | | | |
| **Pulvinar** |  | |  |  |  | |  | | | | |
| **Left** | 1,303.41(411.44) | | 1,492.11(415.78) | 1,369.27(230.93) | .754 | | .390 | | | | |
| **Right** | 1,203.16(385.99) | | 1,427.88(391.21) | 1,238.96(167.12) | 1.706 | | .199 | | | | |
| **Intralaminar** |  | |  |  |  | |  | | | | |
| **Left** | 380.33(91.88) | | 416.73(118.53) | 352.20(50.65) | .311 | | .580 | | | | |
| **Right** | 352.68(87.12) | | 403.74(116.63) | 340.82(62.68) | .990 | | .326 | | | | |
| **MD** |  | |  |  |  | |  | | | | |
| **Left** | 827.26(125.73) | | 932.11(157.36) | 911.45(135.99) | 6.637 | | .014 | | | | |
| **Right** | 844.43(150.33) | | 924.98(147.50) | 931.57(81.13) | 3.519 | | .068 | | | | |

Mean (Standard Deviation).

AV = anteroventral; LD = laterodorsal; LGN: lateral geniculate; LP = lateral posterior; MD = mediodorsal; MGN = medial geniculate; VA = ventral anterior; VLa = ventral lateral anterior; VLp = ventral lateral posterior; VM = ventromedial; VPL = ventral posterolateral.

| **Supplementary Table 7. Group Differences in Hypothalamic Subregional Volumes** | | | | | |
| --- | --- | --- | --- | --- | --- |
|  | ***C9orf72***  **(*n* = 16)** | **Non-carriers**  **(*n* = 28)** | ***GRN (n=4)*** | ***F*** | ***p*** |
| **Hypothalamus** |  |  |  |  |  |
| **Left** | 382.82(30.84) | 401.44(39.94) | 392.90(31.94) | 3.077 | .087 |
| **Right** | 370.19(33.25) | 379.18(36.70) | 367.61(37.52) | .565 | .457 |
| **Anterior Inferior** |  |  |  |  |  |
| **Left** | 19.18(3.33) | 18.97(4.07) | 16.22(2.03) | .003 | .959 |
| **Right** | 18.36(3.92) | 17.02(5.43) | 17.97(3.79) | 1.169 | .286 |
| **Anterior Superior** |  |  |  |  |  |
| **Left** | 24.26(3.31) | 23.25(4.33) | 25.69(3.46) | .290 | .593 |
| **Right** | 25.12(3.66) | 24.45(3.08) | 25.40(1.43) | .585 | .449 |
| **Posterior** |  |  |  |  |  |
| **Left** | 114.51(16.58) | 114.00(15.01) | 111.48(11.99) | .462 | .501 |
| **Right** | 107.99(16.22) | 104.31(16.38) | 105.28(18.32) | .513 | .478 |
| **Tuberal Inferior** |  |  |  |  |  |
| **Left** | 119.22(11.46) | 130.67(16.46) | 119.49(12.58) | 8.015 | .007 |
| **Right** | 113.20(9.33) | 123.74(14.00) | 112.06(11.44) | 6.796 | .013 |
| **Tuberal Superior** |  |  |  |  |  |
| **Left** | 105.65(8.55) | 114.55(14.60) | 120.02(7.92) | 6.393 | .016 |
| **Right** | 105.52(9.61) | 109.66(12.67) | 106.90(4.20) | 1.692 | .201 |

| \| **Supplementary Table 8. Partial correlations between metabolic functions and cortical regions displaying abnormal volumes, controlling for group membership** \| \| \| \| \| \| \| --- \| --- \| --- \| --- \| --- \| --- \| \|  \| **Percent Fat (AM)** \| **Android Gynoid Ratio** \| **VAT Area (cm2)** \| **Total Fat Mass (g)** \| **Total Lean Mass (g)** \| \| **Orbitofrontal** \|  \|  \|  \|  \|  \| \| **Right** \| .083 \| .139 \| -.085 \| .11 \| .279 \| \| **VMPFC** \|  \|  \|  \|  \|  \| \| **Right** \| .071 \| .018 \| -.018 \| .08 \| .173 \| \| **Motor** \|  \|  \|  \|  \|  \| \| **Left** \| -.073 \| .046 \| -.215 \| -.179 \| .32 \| \| **Frontal Pole** \|  \|  \|  \|  \|  \| \| **Left** \| -.112 \| -.472* \| -.374 \| .015 \| -.478* \| \| **Right** \| -.181 \| -.562** \| -.422 \| -.135 \| -.522* \| \| **Medial Temporal** \|  \|  \|  \|  \|  \| \| **Left** \| -.056 \| .434* \| .222 \| -.008 \| .566** \| \| **Lateral Temporal** \|  \|  \|  \|  \|  \| \| **Right** \| -.013 \| .227 \| -.033 \| -.051 \| .396 \| \| **Medial Parietal** \|  \|  \|  \|  \|  \| \| **Right** \| .017 \| .311 \| .069 \| -.034 \| .375 \| \| **Anterior Cingulate** \|  \|  \|  \|  \|  \| \| **Left** \| .149 \| .128 \| .038 \| .086 \| .246 \| \| **Middle Cingulate** \|  \|  \|  \|  \|  \| \| **Right** \| .119 \| .377 \| .167 \| .022 \| .438* \| \| **Posterior Insula** \|  \|  \|  \|  \|  \| \| **Right** \| -.155 \| -.018 \| -.372 \| -.241 \| .148 \|   **p* ≤.01; ***p* ≤.001  AM = Age matched; VAT = visceral adipose tissue.   \| **Supplementary Table 9. Partial correlations between subcortical volumes and metabolic functions, controlling for group membership** \| \| \| \| \| \| \| --- \| --- \| --- \| --- \| --- \| --- \| \|  \| **Percent Fat (AM)** \| **Android Gynoid Ratio** \| **VAT Area (cm2)** \| **Total Fat Mass (g)** \| **Total Lean Mass (g)** \| \| **Pallidum** \|  \|  \|  \|  \|  \| \| **Left** \| -.033 \| .089 \| -.122 \| -.031 \| .245 \| \| **Accumbens** \|  \|  \|  \|  \|  \| \| **Right** \| .167 \| .314 \| .113 \| .116 \| .399 \| \| **Amygdala** \|  \|  \|  \|  \|  \| \| **Right** \| -.058 \| .195 \| -.016 \| -.07 \| .444* \| \| **Caudate** \|  \|  \|  \|  \|  \| \| **Left** \| .057 \| .042 \| -.019 \| -.022 \| .244 \| \| **Right** \| .036 \| .071 \| -.011 \| -.044 \| .228 \|   **p* ≤.01. AM = Age matched; VAT = visceral adipose tissue.   \| **Supplementary Table 10. Partial correlations between thalamic subregional volumes and metabolic functions, controlling for group membership** \| \| \| \| \| \| \| --- \| --- \| --- \| --- \| --- \| --- \| \|  \| **Percent Fat (AM)** \| **Android Gynoid Ratio** \| **VAT Area (cm2)** \| **Total Fat Mass (g)** \| **Total Lean Mass (g)** \| \| **LGN** \|  \|  \|  \|  \|  \| \| **Right** \| .106 \| .032 \| -.131 \| .105 \| .107 \| \| **VLp** \|  \|  \|  \|  \|  \| \| **Left** \| .154 \| .33 \| .229 \| .12 \| .517* \| \| **MD** \|  \|  \|  \|  \|  \| \| **Left** \| .122 \| -.005 \| -.227 \| .019 \| .23 \|   **p* ≤.01. AM = Age matched; LGN: lateral geniculate; MD = mediodorsal; VAT = visceral adipose tissue; VLp = ventral lateral posterior.   \| **Supplementary Table 11. Partial correlations between hypothalamic subregional volumes and metabolic functions, controlling for group membership** \| \| \| \| \| \| \| --- \| --- \| --- \| --- \| --- \| --- \| \|  \| **Percent Fat (AM)** \| **Android Gynoid Ratio** \| **VAT Area (cm2)** \| **Total Fat Mass (g)** \| **Total Lean Mass (g)** \| \| **Tuberal Inferior** \|  \|  \|  \|  \|  \| \| **Left** \| -.082 \| -.012 \| -.235 \| -.184 \| .154 \| \| **Right** \| -.149 \| .1 \| -.139 \| -.196 \| .117 \| \| **Tuberal Superior** \|  \|  \|  \|  \|  \| \| **Left** \| .088 \| -.002 \| -.171 \| -.031 \| .055 \|   AM = Age matched; VAT = visceral adipose tissue. |
| --- | --- | --- | --- | --- | --- | --- | --- | --- | --- | --- | --- | --- | --- | --- | --- | --- | --- | --- | --- | --- | --- | --- | --- | --- | --- | --- | --- | --- | --- | --- | --- | --- | --- | --- | --- | --- | --- | --- | --- | --- | --- | --- | --- | --- | --- | --- | --- | --- | --- | --- | --- | --- | --- | --- | --- | --- | --- | --- | --- | --- | --- | --- | --- | --- | --- | --- | --- | --- | --- | --- | --- | --- | --- | --- | --- | --- | --- | --- | --- | --- | --- | --- | --- | --- | --- | --- | --- | --- | --- | --- | --- | --- | --- | --- | --- | --- | --- | --- | --- | --- | --- | --- | --- | --- | --- | --- | --- | --- | --- | --- | --- | --- | --- | --- | --- | --- | --- | --- | --- | --- | --- | --- | --- | --- | --- | --- | --- | --- | --- | --- | --- | --- | --- | --- | --- | --- | --- | --- | --- | --- | --- | --- | --- | --- | --- | --- | --- | --- | --- | --- | --- | --- | --- | --- | --- | --- | --- | --- | --- | --- | --- | --- | --- | --- | --- | --- | --- | --- | --- | --- | --- | --- | --- | --- | --- | --- | --- | --- | --- | --- | --- | --- | --- | --- | --- | --- | --- | --- | --- | --- | --- | --- | --- | --- | --- | --- | --- | --- | --- | --- | --- | --- | --- | --- | --- | --- | --- | --- | --- | --- | --- | --- | --- | --- | --- | --- | --- | --- | --- | --- | --- | --- | --- | --- | --- | --- | --- | --- | --- | --- | --- | --- | --- | --- | --- | --- | --- | --- | --- | --- | --- | --- | --- | --- | --- | --- | --- | --- | --- | --- | --- | --- | --- | --- | --- | --- | --- | --- | --- | --- | --- | --- | --- | --- | --- | --- | --- | --- | --- | --- | --- | --- | --- | --- | --- | --- | --- | --- | --- | --- | --- | --- | --- | --- | --- | --- | --- | --- | --- | --- | --- | --- | --- | --- |
